# Progressive Vascular Abnormalities in the Aging 3xTg-AD Mouse Model of Alzheimer’s Disease

**DOI:** 10.3390/biomedicines10081967

**Published:** 2022-08-13

**Authors:** Amandine Jullienne, Ryan Quan, Jenny I. Szu, Michelle V. Trinh, Erik J. Behringer, Andre Obenaus

**Affiliations:** 1 Department of Pediatrics, University of California, Irvine, CA 92697, USA; 2 Department of Basic Sciences, Loma Linda University, Loma Linda, CA 92350, USA

**Keywords:** Alzheimer’s disease, vessels, branching, transgenic mouse, fractals

## Abstract

Vascular dysfunction and structural abnormalities in Alzheimer’s disease (AD) are known to contribute to the progression of the pathology, and studies have tended to ignore the role of the vasculature in AD progression. We utilized the 3xTg-AD mouse model of AD to examine individual cerebral vessels and the cortical vascular network across the lifespan. Our vessel painting approach was used to label the entire cortical vasculature, followed by epifluorescence microscopy. The middle cerebral artery (MCA) tree was assessed with confocal microscopy, and a new method was developed to assess branching patterns as a measure of aging-related changes. We found that vascular remodeling was profoundly altered at 4–6 months of age, when the 3xTg-AD mouse is known to transition to cognitive impairment and Aβ deposition in both sexes. Analysis of vascular features (density, junctions, length) of the MCA territory highlighted sex-dependent differences across the 3xTg-AD mouse lifespan, with no alterations in branching patterns. Our current cerebrovascular angioarchitectural analyses demonstrate progressive alterations in individual cortical vessels, as well as in the vascular network of the cortex. These new findings advance our understanding of brain anatomy and physiology in the 3xTg-AD mouse, while potentially identifying unique diagnostic signatures of AD progression.

## 1. Introduction

Alzheimer’s disease (AD) is the leading form of neurodegenerative dementia, currently impacting ~6.5 million people in the United States alone [1]. A primary contribution to AD pathology entails vascular dysfunction that encompasses intracranial arteries/arterioles [2,3,4], capillaries [5], and more recently, pericytes [6,7]. Consequently, reduced cerebral blood flow [3] and brain oxygenation [4] emerge in tandem with the increased presence of white matter hyperintensities [2,8] in the AD brain. The cerebral hypoperfusion that precedes and accompanies AD underlies hypoxia and a generalized reduction in cellular adenosine triphosphate (ATP) synthesis in the brain which, in turn, results in dysfunctional protein synthesis and degradation; ion channel, receptor, and pump dysfunction; and signaling cascade impairment [9,10]. Thus, over time, accumulated proteinopathy as toxic amyloid-β (Aβ) plaques and neurofibrillary tangles composed of hyperphosphorylated tau emerge as overt biomarkers of AD [11]. Further, the cellular impact of Aβ plaques and neurofibrillary tangles, e.g., imbalance among action potential firing and synaptic plasticity [12], is exacerbated by impaired clearance mechanisms via cerebral venules, lymphatic vessels, and the glymphatic system [13].

Recent evidence demonstrates that cerebrovascular resistance predicts progression of AD etiology from amyloid-independent brain atrophy and mild cognitive impairment (MCI) to amyloidosis that leads to dementia [14,15]. Thus, the early structural and functional remodeling of cerebral vessels leads to impaired blood flow delivery and perfusion of the brain, resulting in AD-type dementia [5,15]. However, as a viable path to enhancing diagnosis and therapy, clear signatures of vascular angioarchitectural alterations accompanying AD pathogenesis remain unclear. The cerebral circulation engages with blood flow control, vascular permeability, and angiogenesis to optimally perfuse the brain for healthy cognition throughout life. In AD, this careful orchestration of vascular function is perturbed.

Recent studies have examined the cerebral angioarchitecture using methods such as corrosion casting and micro-computed tomography [16], as well as imaging of perivascular Aβ deposits paired with miRNA expression [17]. Accordingly, emerging evidence in mouse models recapitulating human familial AD (e.g., 3xTg-AD, 5XFAD) suggest that cerebrovascular remodeling and/or degeneration occurs before the onset of classical AD hallmarks, such as the presence of Aβ plaques and neurofibrillary tangles [10,16,18]. The need for focused, high-quality imaging of individual cerebral vessels and the vascular network during the development of AD is of paramount importance. Additionally essential is determining how the vasculature responds to AD within the context of biological sex, particularly when there are early cognitive deficits without overt pathology or onset of amyloidosis.

Employing our novel vessel painting approach to study cortical regions in AD model mice, we tested the hypothesis that cerebrovascular structural profiles are modified by the onset of AD pathology in both males and females. We temporally examined aging 3xTg-AD mice as they progressed from young (1–3 months), cognitively impaired (4–6 months), showing the presence of extracellular amyloid deposits (Aβ; 7–9 months), and manifesting Aβ plaques in combination with neurofibrillary tangles (Aβ-NFT; 12–15 months) [18,19]. A key advantage of the 3xTg-AD mouse model is that homozygous offspring develop phases of cumulative AD pathology over a measurable, phased aging process that are relatively absent in the originating background strain (B6129SF2/J; ≤15 months), with progressive alterations in neurovascular coupling for cerebral blood flow [20], cognitive function [21], and learning/memory [19,22,23]. In brief, we found that vascular remodeling (density, branching, length, and complexity) was most profoundly altered between 4 and 9 months in both biological sexes, whereby older conditions did not differ significantly relative to the younger, pre-AD groups. Analysis of the middle cerebral artery (MCA) territory highlighted sex-dependent differences as the 3xTg-AD mouse ages. We also introduce an advancement in our analytical methods for examining MCA branching patterns. In summary, we believe that our current structural analyses of the cerebrovasculature further advance our understanding of brain anatomy and physiology while potentially identifying a unique diagnostic fingerprint for the progression of AD.

## 2. Materials and Methods

### 2.1. 3xTg-AD Mice

All animal care and use experimental protocols for this study were approved by the Institutional Animal Care and Use Committees of Loma Linda University and the University of California-Irvine and performed in accordance with the National Research Council’s “Guide for the Care and Use of Laboratory Animals” (8th Edition, 2011). Experiments were performed using male and female 3xTg-AD mice that were inbred from homozygous breeding pairs obtained from the Jackson Laboratory (Wilmington, MA, USA). We chose the 3xTg-AD study model [(B6;129-Tg (APP-Swe, tauP301L) 1Lfa Psen1tm1Mpm/Mmjax]; Mutant Mouse Resource and Research Center (MMRRC) stock #034830] that shows progressive microvascular degeneration [16] concomitant with reduced neurovascular coupling underlying cerebral blood flow [20], synaptic dysfunction [21], and learning/memory deficits [19,22,23]. In accordance with cumulative AD pathology, we categorized the mice into four groups as young (1–3 months; males, *n* = 8; females, *n* = 8), cognitive impairment (4–6 months; males, *n* = 10; females, *n* = 12), presence of extracellular amyloid-β plaques (Aβ; 7–9 months; males, *n* = 9; females, *n* = 9), and presence of Aβ and neurofibrillary tangles composed of tau (Aβ-NFT; 12–15 months; males, *n* = 11; females, *n* = 10) [18,19]. All animals were housed on a 12:12 h light-dark cycle at 22–24 °C with fresh water and food available ad libitum.

### 2.2. Vessel Painting

Our vessel painting technique is based on the ability of the fluorescent dye 1,1′-dioctadecyl-3,3,3′3′-tetramethylindocarbocyanine perchlorate (DiI, Life Technologies, Carlsbad, CA, USA) to bind to lipid membranes. Our previously published protocol was modified for this study [24]. Mice were intraperitoneally injected with heparin and sodium nitroprusside and 5 min later, were anesthetized with an intraperitoneal injection of ketamine (90 mg/kg) and xylazine (10 mg/kg). Vessel painting was performed by injecting a solution of DiI (0.3 mg/mL in PBS containing 4% dextrose, total volume of 500 µL) into the left ventricle, followed by a 10 mL PBS flush and a 20 mL 4% PFA perfusion, using a peristaltic pump (8.4 mL/min). The brains were extracted, post-fixed in 4% PFA for 24 h, washed, and stored at 4 °C in PBS until imaging. Successfully vessel painted brains were selected if they showed uniform pink staining and excellent staining of large and small vessels on the cortical surface, as illustrated previously [24]. In this study, 77.9% (60/77) of the brains were successfully stained and analyzed.

### 2.3. Imaging and Analysis of Vessel Painted Brains

The brains were imaged using a fluorescence microscope (Keyence BZ-X810, Keyence Corp, Osaka, Japan). Axial images of the entire brain were acquired at 2× magnification using the Z-stack feature (~42 images, step size 25.2 µm). The proximal aspect of the left middle cerebral artery (MCA) was also imaged for each sample using confocal microscopy (10× magnification, Z-stack 30 images, step size 1.51 μm, Olympus FV3000, Olympus Scientific, Waltham, MA, USA).

Classical vessel analysis was performed by using the Angiotool software (Version 0.6a), allowing for measures of vessel density, length, and number of junctions [22], and the ImageJ plugin “FracLac” was used to analyze vascular complexity through fractal analysis [23]. Details of this analysis protocol have been previously published [24].

### 2.4. Branching Analysis of the Middle Cerebral Artery (MCA)

**Figure 1:**
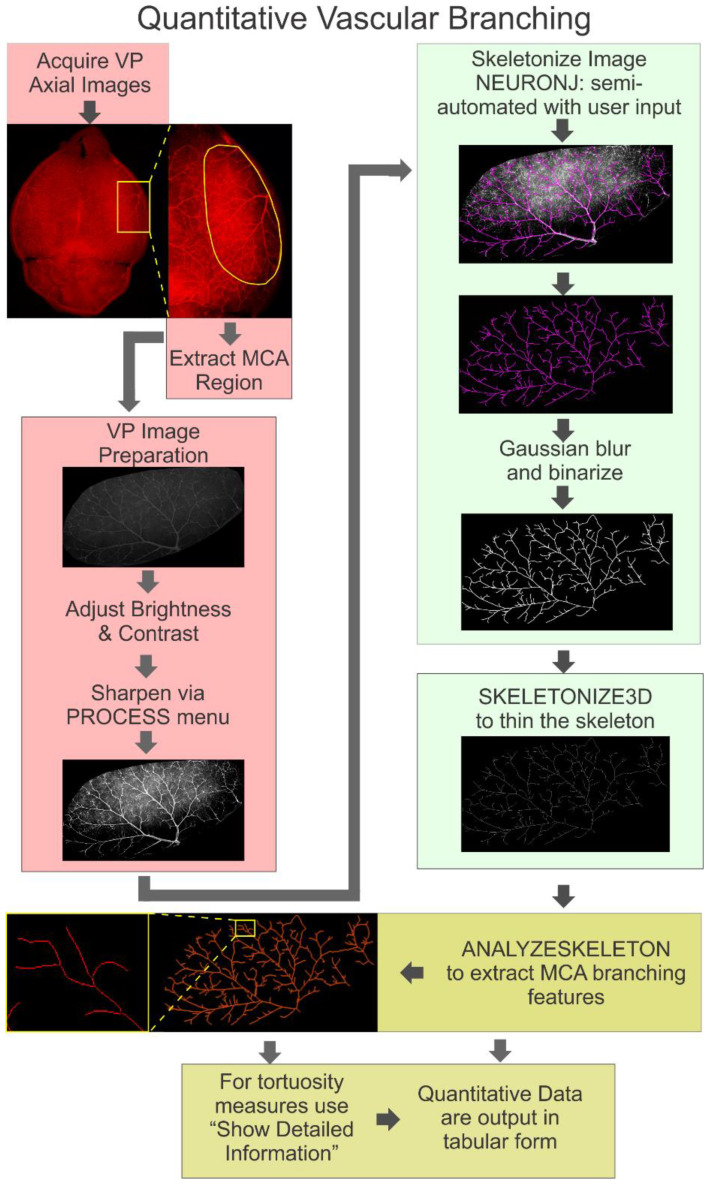
Workflow of middle cerebral artery (MCA) skeletonization. The skeletonization workflow consisted of five main stages: image acquisition, image preparation, NeuronJ tracing, skeleton thinning, and extraction of branching features. Fluorescent microscopy acquired images from vessel-painted brains (see Figure 2a). Images were then adjusted for brightness, contrast, and sharpened to provide optimal visualization of the MCA; this is crucial for optimal subsequent MCA skeletonization in NeuronJ. Then, using the Skeletonize3D application, a Gaussian blur is applied followed by binarization. Then, the resultant NeuronJ-derived skeleton is further thinned. The last step is the quantification of MCA vascular branching features using AnalyzeSkeleton. In the resultant images, pixels from the skeleton are color-coded according to their classifications as either branches (orange), junctions (purple), or end points (blue).

Vascular branching and its associated complexity have been reported to be related to human AD [25], but this association has not been overtly studied in animal models of AD. Thus, to further extend our understanding of vascular branching in the 3xTg-AD model from our acquired vessel painting data, we sought to develop a robust and efficient quantitative protocol from existing available tools. All branching tools utilized in this protocol were derived from Fiji (Version 1.49v, National Institute of Health, Bethesda, MA, USA) and currently available Java plugins (ImageJ can also be utilized). Herein, we outline a protocol that estimates quantitative data from the branching patterns and from MCA vascular networks. This protocol is summarized in Figure 1. There are three primary operations: (1) preparing the vessel painting images and extraction of the MCA territory, (2) MCA tracing and skeletonization, and (3) quantification of MCA parameters, including tortuosity.

#### 2.4.1. Image Preparation

Acquisition of axial images of the MCA territory from vessel painted brains was performed as detailed in Section 2.3. Using axial Z-stack projections (~42 images) of the brain, the entire MCA territory was extracted using Fiji’s polygon selection tool. After extraction, each MCA image was optimized using standard Fiji tools for brightness and contrast to visually enhance the vascular tree (Figure 1, raw, optimal vs. non-optimal). This is a crucial step, and care should be taken to optimize the images, as this will facilitate future skeletonization processes, as well as the quality of the quantitative results. We observed that Fiji’s brightness and contrast “Auto” feature provided adequate visualization of the MCA from our standard acquisitions. The lower bound of the brightness setting may also be increased later to filter out background signals. Since pixel brightness can be heterogeneous within the MCA itself, care should be taken in ensuring that “darker” pixels of the artery are not excluded during thresholding. After brightness and contrast are optimized, the image is sharpened using Fiji’s built-in Process tools. The adjusted settings are saved by pressing “apply,” and the final contrasted image is saved in an 8-bit grayscale format for compatibility with other plugins (see Figure 1).

#### 2.4.2. Skeletonization of the MCA

After image preparation, the Fiji plugin NeuronJ (Version 1.4.3) was utilized [26]. This plugin was developed to skeletonize and quantify neurons and their neurites. Here, we have repurposed NeuronJ to skeletonize the MCA vascular tree. Based on our vessel painted images, we found that the following parameters can accurately detect the branches of the MCA: neurite appearance = bright, Hessian smoothing scale = 2, cost weight factor = 0.7, snap window size = 9 × 9, path-search window size = 2500 × 2500, tracing smoothing range = 5, tracing subsampling factor = 5, and line width = 3. Future users should optimize these parameters for their samples. At this point, the user can opt to extract basic quantitative measures from skeletonized vessels including total length, mean, standard deviation, and minimum and maximum tracing lengths. Other quantitative features are available in NeuronJ, but they were not utilized in this protocol. Upon completion of semi-automated tracing of the MCA, “snapshot” images of the skeleton overlay and its isolated skeleton are saved as RGB files (Figure 1).

After skeletonization of the MCA vascular tree, the next step is to prepare the image for extraction of quantitative vascular branching data. The RGB MCA skeleton tracing derived from NeuronJ was converted into an 8-bit image, Gaussian blurred (Sigma = 1), and binarized. The resultant image was thinned using the Fiji plugin Skeletonize3D (Version 1.0.2) [27,28,29], which provides an appropriate input file for quantification using AnalyzeSkeleton (Version 3.4.2) [27].

#### 2.4.3. Quantification of Skeletonized Vascular Tree

AnalyzeSkeleton was used to open the finalized binary MCA skeleton. The plugin authors note that this plugin will tag all the 2D pixels (or 3D voxels) in a skeletonized image and can identify and color code each pixel as either an end point (blue), junction (purple), or slab (orange) (Figure 1) [27]. Several options are available to the user, but for our analyses, we selected the following: prune cycle method = none, results and output = show detailed info, display labeled skeletons. After opening the isolated NeuronJ tracing in Fiji, the entire process described above can be automated using the following macro (see sample in Supplement File S1).

In cases where many vascular loops are present, the user may also opt to use the latest version of the plugin that allows for the pruning of loops. In our analysis, we did not use loop pruning, as loops were uncommon, and in order to preserve the structure of the skeleton.

The plugin performs the analysis with an output of a results table showing the number of branches, and the number of voxels that are end-points, slabs, or junctions. It also counts the number of junctions, number of junctions with 3 or 4 branches, and average and maximum length of the branches. If the user opts for additional information by checking the “show detailed information,” box, the table will include the following: skeleton ID (if more than one skeleton in an image was analyzed), branch length, 3D coordinates of the branch extremes (V1 and V2 vertices), and the Euclidean distance between the branch extremes.

While tortuosity measures are not automatically computed, they can be generated by taking the ratio of the branch length to the Euclidean distance between branch extremes using Excel or other data analysis software. Larger values (T > 1) indicate more tortuous vessels. In our study, the tortuosity of collateral vessels (between MCA and PCA/ACA) was analyzed using this approach.

### 2.5. Statistical Analyses

Individuals performing the analyses were blinded to genotype and sex. All data were summarized in Microsoft Excel prior to statistical testing. Statistical analyses were performed using GraphPad Prism 9 software (GraphPad Prism, San Diego, CA, USA). Box and whiskers graphs illustrated median, first, and third quartile maximum and minimum values. All other bar and line graphs are shown as mean ± standard error of the mean. Data grouping males and females passed the normality tests (D’Agostino and Pearson test, Shapiro–Wilk test) and were analyzed with one-way ANOVA and post hoc Tukey’s multiple comparisons test. Data separating males and females were analyzed with 2-way ANOVA and post hoc Sidak’s multiple comparisons test. Statistical significance was designated as *p* < 0.05.

## 3. Results

### 3.1. Classical Cortical Vascular Networks of 3xTg-AD Mice Are Subtly Remodeled with Age

**Figure 2:**
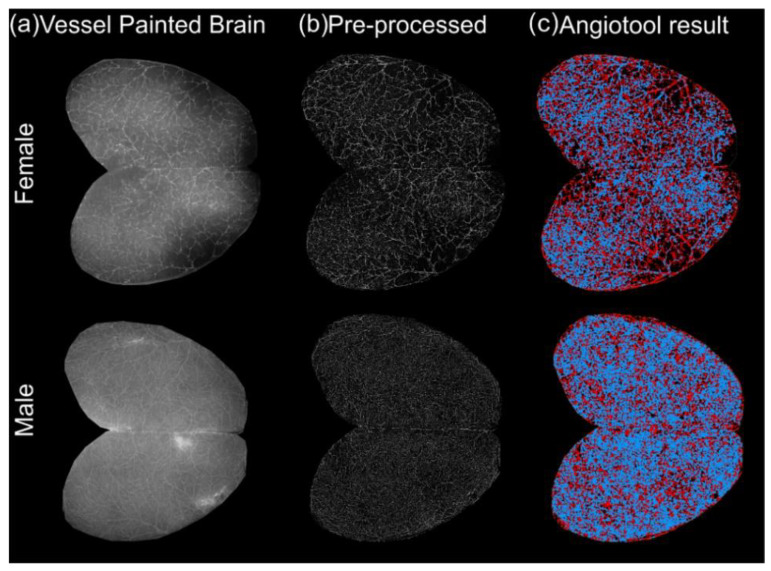
Quantitative workflow for axial cortical surface vascular assessments in 1 to 3-month-old 3xTg-AD mice. (**a**) Exemplar vessel painted brains from male and female 3xTg-AD mice. (**b**) Vessel painting images are pre-processed by binarizing images and then subjected to haze reduction using the Keyence BZ-X800 Analyzer. (**c**) The pre-processed images are then analyzed using Angiotool, which results in output images where vessels are represented in red and junctions/branching points in blue.

The cortical vasculature of the aging 3xTg-AD mice acquired using fluorescence microscopy allows for visualization of vessels up to ~1 mm in depth (Figure 2). After acquisition of vessel painted images (Figure 2a) and haze reduction pre-processing (Figure 2b), data were processed for classical angiographic measures using the Angiotool software (Figure 2c).

**Figure 3:**
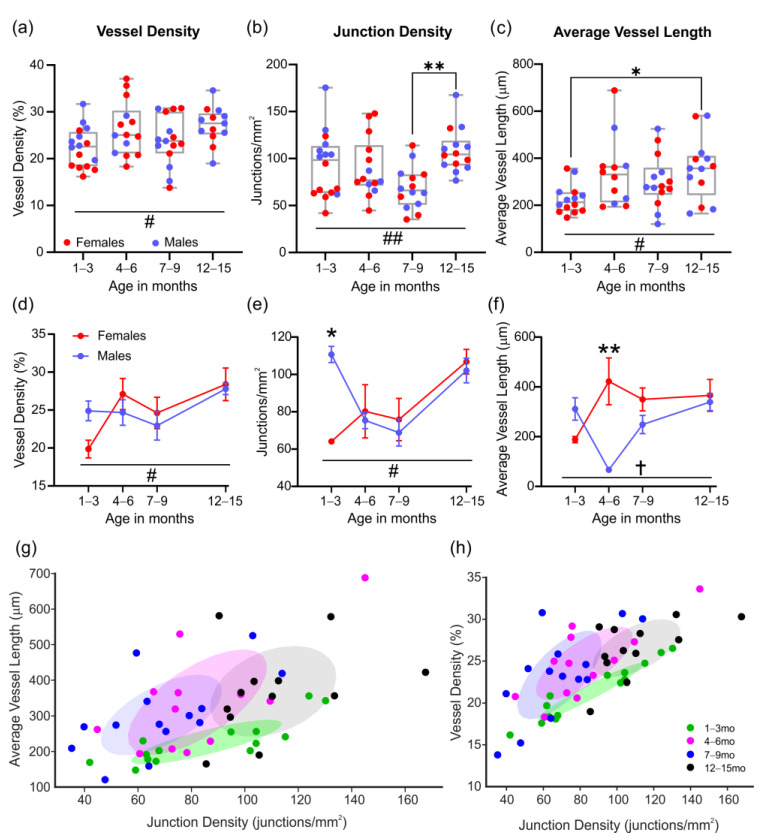
Vascular network features from the cortex in aging 3xTg-AD mice. Vessel density (**a**), junction density (**b**), and average vessel length (**c**) for male and female mice with increasing age. There is an increase in junction density with age (**b**) that is associated with increasing vessel length (**c**). Male and female differences were observed in vessel density (**d**), junction density (**e**), and average vessel length (**f**). Scatter plots showing the relationship between junction density and average vessel length (**g**) or vessel density (**h**), with ellipses showing positive covariance for each age group. * *p* < 0.05, ** *p* < 0.01. # shows a significant effect of age with # *p* < 0.05, ## *p* < 0.01; † shows a significant effect of sex with † *p* < 0.05. Data shown in (**d**–**f**) as mean ± SEM.

Over the natural lifespan of male and female 3xTg-AD mice, we did not observe any significant changes in vascular density between ages, despite a slow progressive increase over time (# *p* = 0.038, one-way ANOVA, Figure 3a). Junction density was significantly different between 7 to 9 and 12 to 15-month-old 3xTg-AD mice (** *p* = 0.003, one-way ANOVA), in part due to a group-wide reduction in junctions at 7–9 months (Figure 3b). The average vessel length was also significantly increased between 1 to 3-month and 12 to 15-month-old 3xTg-AD mice (* *p* = 0.029, one-way ANOVA, Figure 3c).

When the data were dichotomized by sex, two key differences were observed. In male 3xTg-AD mice at 1–3 months of age, there were no significant differences in vessel density (Figure 3d), but a significant increase in the number of junctions was observed relative to female 3xTg-AD mice (* *p* = 0.046, 2-way ANOVA, Figure 3e). By 4–6 months of age, male and female 3xTg-AD mice showed divergent changes in average vessel length, which was decreased in males, and increased in females, with significant differences between the sexes at this age (** *p* = 0.001, 2-way ANOVA, Figure 3f).

When analyzing the relationship between average vessel length and junction density, we observed a more confined distribution in 1 to 3-month-old mice relative to older ages (Figure 3g). The same phenomenon was observed for the relationship between vessel and junction densities (Figure 3h). In both cases, the covariance ellipsoids were positive.

In summary, the classical vascular features of the cortex were not overtly altered over the lifespan of 3xTg-AD mice. Nuances between sexes could be observed, with a higher number of junctions in males early in life, but reduced vessel length later in life compared to females.

### 3.2. Cortical Vascular Complexity in 3xTg-AD Mice

**Figure 4:**
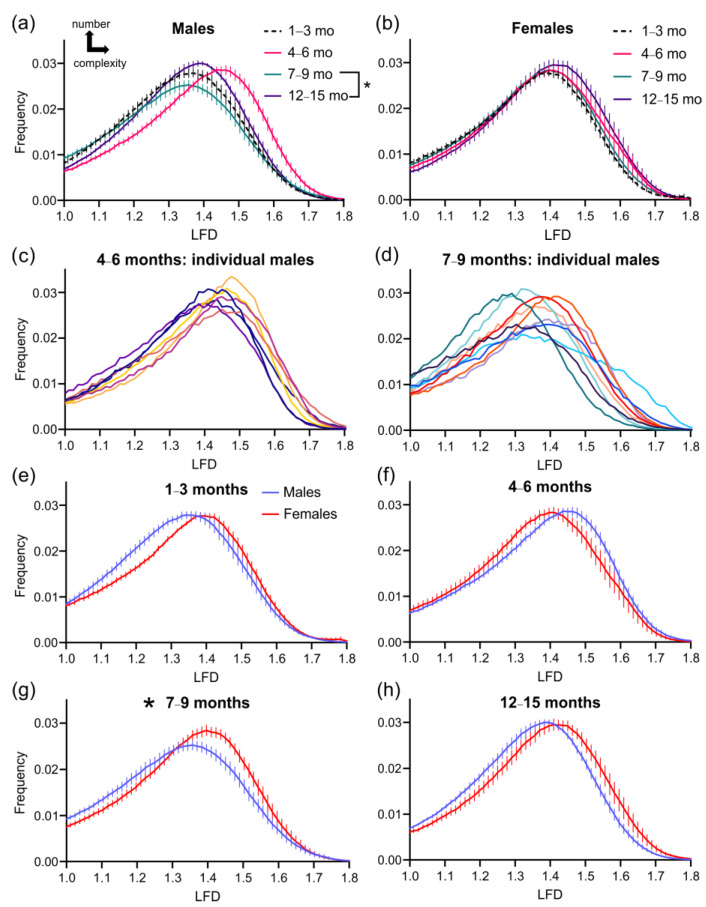
Local fractal dimension (LFD) histograms for vascular complexity. The cortical vessel complexity in aging 3xTg-AD mice illustrates robust differences in males (**a**), but not in females (**b**). Individual LFD histograms in male mice at 4–6 months (**c**) and at 7–9 months of age (**d**) demonstrate increasing variability at later ages. Each line in (**c**) and (**d**) represents an individual mouse. Comparison of LFD histograms between males and females are shown for 1–3 months (**e**), 4–6 months (**f**), 7–9 months (**g**), and 12–15 months (**h**) groups, illustrating the dynamic alterations with time. At 7–9 months of age, there was a significant difference between males and females. * *p* < 0.05 for the skewness and kurtosis values (see Figure 5). Data shown as mean ± SEM.

Vascular complexity was assessed using fractal analyses, as we have done previously [24]. Local fractal dimension (LFD) histograms were generated for each animal and averaged. In male 3xTg-AD mice, there was an increase (rightward shift) in cortical vascular complexity at 4–6 months, but a decreased number of vessels at 7–9 months of age (decreased amplitude, Figure 4a). In female 3xTg-AD mice, there was no overt change, except for a subtle increase in vascular complexity at 12–15 months of age (Figure 4b). A closer examination of each male 3xTg-AD mouse at 4–6 months found little variability in the LFD (Figure 4c), in contrast to the 7–9 month-old male 3xTg-AD mice that exhibited considerable variability, with LFDs ranging from 1.25–1.45 (Figure 4d). When averaged LFD histograms by sex were plotted (Figure 4e–h), only the 7 to 9-month-old 3xTg-AD mice showed significant differences between males and females (Figure 4g).

**Figure 5:**
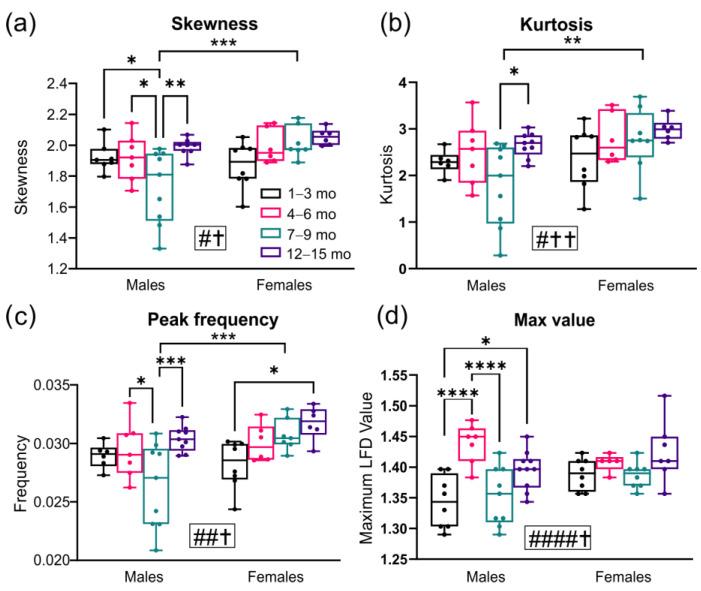
Local fractal dimension (LFD) histogram analyses. Analysis of LFD histograms for skewness (**a**), kurtosis (**b**), peak frequency value (**c**), and maximum LFD values (**d**) in aging male and female 3xTg-AD mice. Males consistently exhibited significant temporal changes in skewness, peak frequency, and maximum LFD value compared to females. The 7–9 month timepoint showed significant differences between males and females, but these differences were not observed at other timepoints. * *p* < 0.05, ** *p* < 0.01, *** *p* < 0.001, **** *p* < 0.0001. # shows a significant effect of age with # *p* < 0.05, ## *p* < 0.01, #### *p* < 0.0001; † shows a significant effect of sex with † *p* < 0.05, †† *p* < 0.01.

LFD histogram features can be quantified for skewness (measure of the asymmetry of the distribution), kurtosis (peakedness), peak LFD frequency, and maximum LFD values (Figure 5). Significant differences between ages in male 3xTg-AD mice were found, with decreasing skewness from 1–9 months, which then reverted to 1 month levels (males 1–3 months vs. 7–9 months, * *p* = 0.044, males 4–6 months vs. 7–9 months, * *p* = 0.047, males 7–9 months vs. 12–15 months, ** *p* = 0.002; 2-way ANOVA, Figure 5a). No differences in skewness were observed among individual female groups, but a significant difference was present between males and females at the 7–9 month timepoint (*** *p* = 0.0006, 2-way ANOVA, Figure 5a). Kurtosis measures in the male were only significantly different between the 7 to 9 and 12 to 15-month-old 3xTg-AD mice (* *p* = 0.016, 2-way ANOVA, Figure 5b), and kurtosis was decreased compared to females at 7–9 months (** *p* = 0.005, 2-way ANOVA, Figure 5b). Peak frequency in the male 3xTg-AD mice was biphasic, with a decrease at 7–9 months of age before returning to the same average values at the other timepoints (males 4–6 months vs. 7–9 months, * *p* = 0.027, 7–9 months vs. 12–15 months, *** *p* = 0.0009; 2-way ANOVA, Figure 5c). In the females, there was a progressive increase in peak frequency with increasing age that was significantly elevated at 12–15 months compared to the 1–3 month-old age group (* *p* = 0.015, 2-way ANOVA, Figure 5c). Again, 7–9 month-old males showed significantly decreased peak frequency compared to females at the same timepoint (*** *p* = 0.0005, 2-way ANOVA, Figure 5c). The maximum LFD value in male 3xTg-AD mice was significantly different across each time epoch (males 1–3 months vs. 4–6 months, **** *p* < 0.00001, 4–6 months vs. 7–9 months **** *p* < 0.00001, 1–3 months vs. 12–15 months * *p* = 0.018; 2-way ANOVA), without any significant changes in females (Figure 5d). In the males, significant elevations at 4–6 months returned at the 7–9 month period to the 1–3 month values, which then slowly increased by 12–15 months, suggesting a dynamic vascular complexity that occurs over the 3xTg-AD lifespan (Figure 5d).

Somewhat surprisingly, there were no significant complexity differences in female 3xTg-AD mice in fractal metrics except for in peak frequency. However, these vessel complexity findings are similar to the classical vascular features, where males principally exhibited vascular alterations compared to more stable female 3xTg-AD mice.

### 3.3. Vascular Measures of the Middle Cerebral Artery

**Figure 6:**
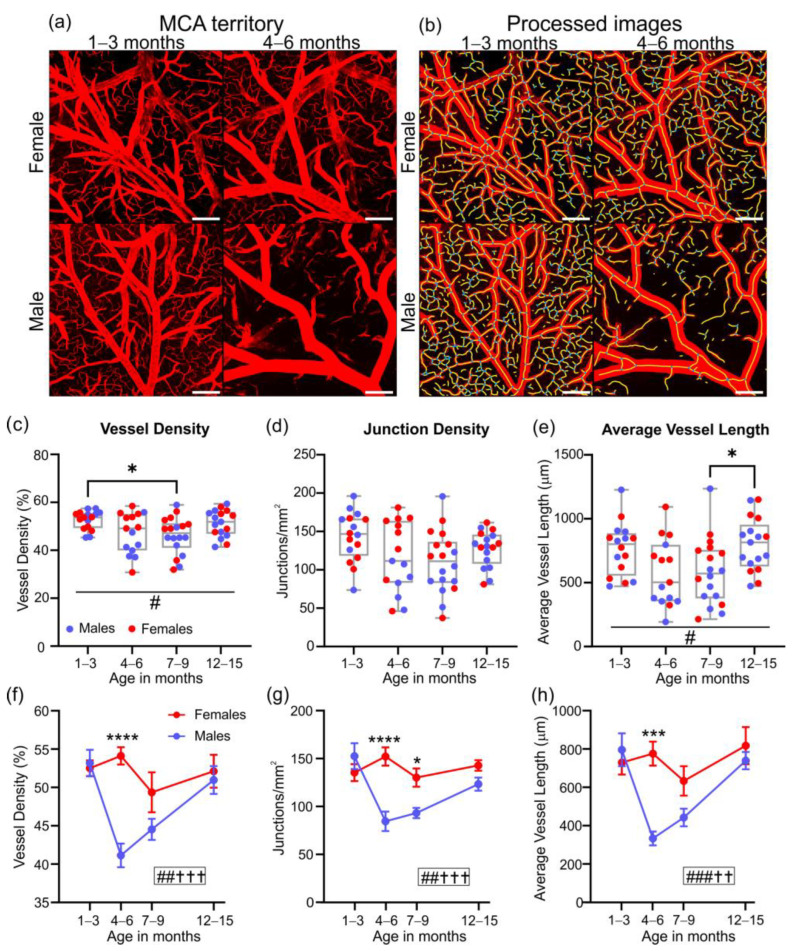
Assessment of the middle cerebral artery (MCA) territory vascular network. Representative MCA confocal images from vessel painted male and female mice at 1–3 and 4–6 months of age (**a**) and after Angiotool processing (**b**), where vessels are in yellow and junctions/branching points are in blue. Scale bars = 200 µm. 3xTg-AD MCA vessel characteristics, including vessel density (**c**), junction density (**d**), and average vessel length (**e**) reveal modest differences with age in vessel density and average vessel length. Male and female MCA vessel differences are shown for vessel density (**f**), junction density (**g**), and average vessel length (**h**), where the 4–6 month timepoint appears as a particularly vulnerable period in male mice. * *p* < 0.05, *** *p* < 0.001, **** *p* < 0.0001. # shows a significant effect of age, with # *p* < 0.05, ## *p* < 0.01, ### *p* < 0.001; † shows a significant effect of sex, with †† *p* < 0.01, ††† *p* < 0.001. Data shown in (**f**–**h**) as mean ± SEM.

A distinct advantage of the vessel painting approach is that it facilitates a closer examination of the vessels, such as the middle cerebral artery (MCA), by confocal microscopy (Figure 6). Exemplar MCA confocal images show reduced vascular density in 4 to 6-month-old males compared to female 3xTg-AD mice at 4–6 months and compared to 1 to 3-month-old mice. These decrements are further borne out in the Angiotool images (Figure 6b). There was a significant decline in MCA vessel density by 7–9 months compared to 1–3 months (* *p* = 0.031, one-way ANOVA, Figure 6c), with no change in vessel densities over time (Figure 6d). The average vessel length was only significantly different between the 7–9 and 12–15 month timepoints (* *p* = 0.045, one-way ANOVA, Figure 6e).

When 3xTg-AD mice were dichotomized by sex, significant differences between males and females were observed, with vessel density significantly reduced at 4–6 months of age in males compared to females (**** *p* < 0.0001, 2-way ANOVA, Figure 6f). A virtually identical difference was apparent in male 3xTg-AD mice at the 4–6 month timepoints for junction density (**** *p* < 0.0001, 2-way ANOVA, Figure 6g) and average vessel length (*** *p* = 0.0001, 2-way ANOVA, Figure 6h).

Thus, as noted above, the vascular features of the MCA in male 3xTg-AD mice are reduced at 4–6 months of age relative to later in life. This suggests that in 3xTg-AD males, increased vascular pruning occurs from 1–3 months to 4–6 months of age with subsequent vessel regrowth with age. We have observed a similar loss and regrowth on the contralateral side in a model of brain trauma [30].

### 3.4. Analysis of the Middle Cerebral Artery Skeleton

**Figure 7:**
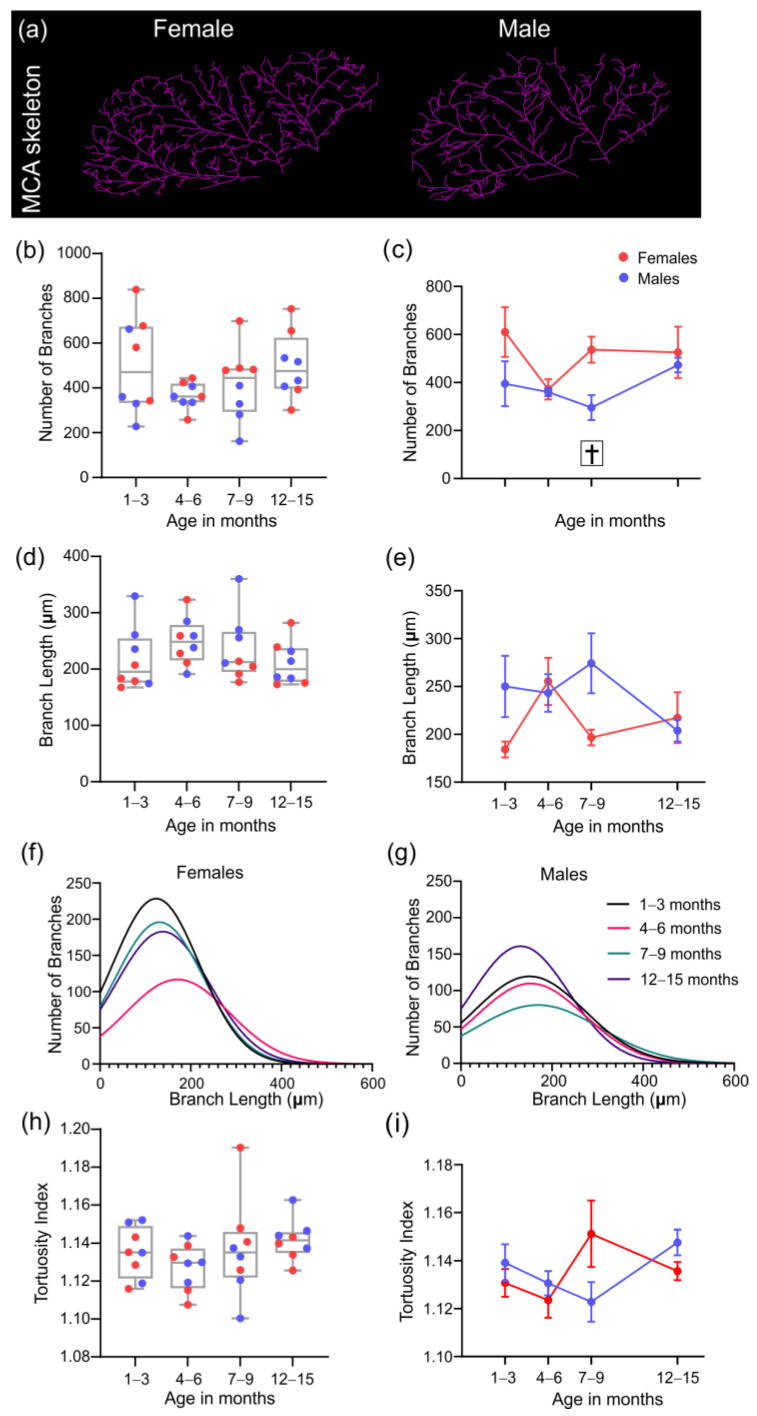
MCA skeleton derived analytic features. Representative images illustrating the MCA skeleton for female and male 3xTg-AD mice at 7–9 months of age (**a**). Total number of MCA branches was not statistically different between age groups (**b**) nor for males and females (**c**). Similarly, there are no differences in average MCA branch length across age groups (**d**) or by sex (**e**). The distribution of the branch length is represented for females (**f**) and males (**g**). The average tortuosity index of the MCA branches is shown for all age groups (**h**) and separated by sex (**i**). † indicates a significant effect of sex, with † *p* < 0.05. Data shown in (**c**,**e**,**i**) as mean ± SEM. Note that comparisons among males versus females at the 7–9 month timepoint are indicated by *p* = 0.088, *p* = 0.084, and *p* = 0.062 for panels (**c**,**e**,**i**), respectively.

The MCA skeleton of 4 mice per group was analyzed using our new protocol designed to characterize the skeleton of the vascular tree. Representative images illustrating the MCA skeleton for female and male 3x-Tg-AD mice at 7–9 months of age are shown in Figure 7a. Quantitatively, the number of vessel branches was not significantly different between age groups (Figure 7b), but there was an effect of sex across ages († *p* = 0.015, 2-way ANOVA, Figure 7c). Females tend to have a greater number of MCA branches when compared to males, especially at 7–9 months (*p* = 0.088, 2-way ANOVA, Figure 7c). No significant differences in average branch length were observed between ages (Figure 7d) nor sexes (Figure 7e).

The distributions of vessel branch length were then examined in males and females across the lifespan. There was an increased number of branches in females at the 1–3, 7–9, and 12–15 month timepoints (Figure 7f) compared to males (Figure 7g). Figure 7f–g also reveals that the majority of the branches measure about 120–160 μm for both sexes. Finally, the tortuosity of the MCA vessel branches did not show any differences across lifespan (Figure 7h). There is a trending difference at 7–9 months between sexes, where the tortuosity index is increased for females (*p* = 0.062, 2-way ANOVA, Figure 7i).

### 3.5. Analysis of the Collateral Vessels in the Middle Cerebral Artery Vascular Tree

**Figure 8:**
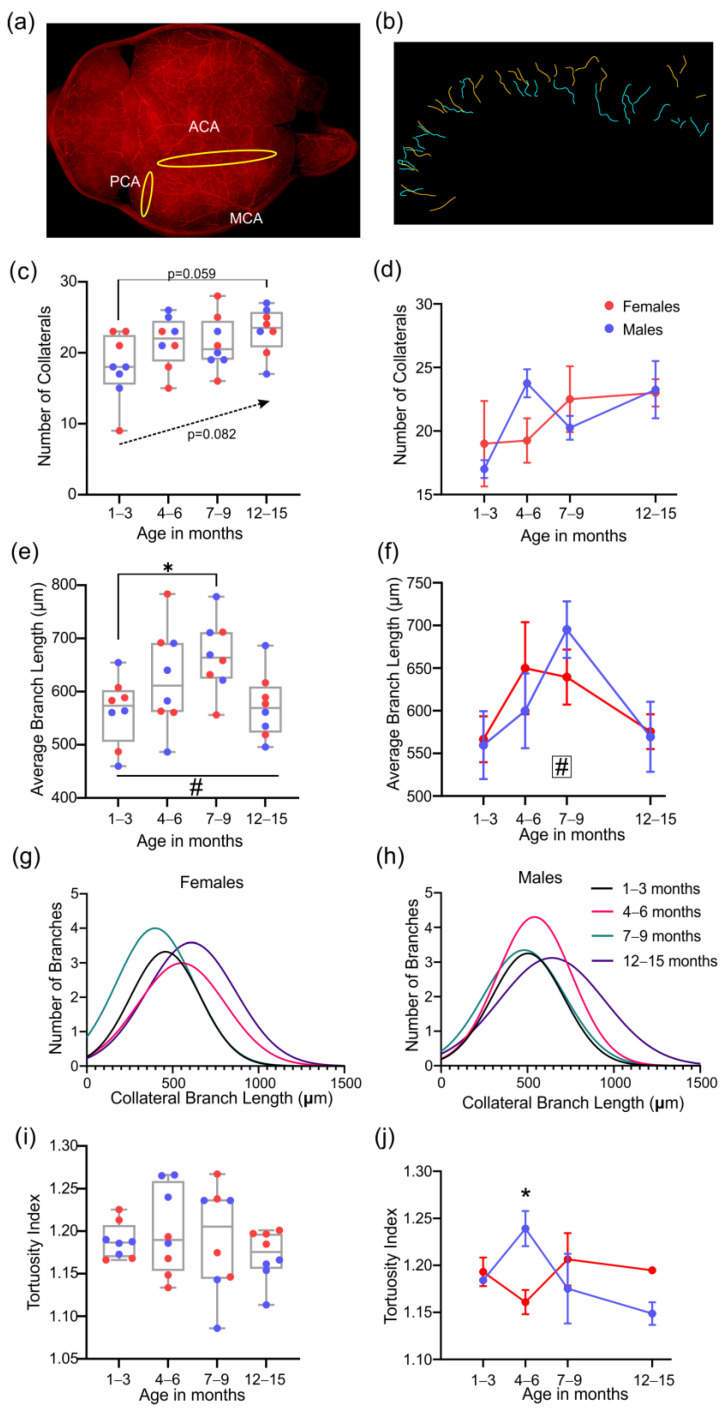
Collateral vessels between MCA and ACA/PCA. (**a**) Representative images illustrating the location of the collaterals and the region of anastomosis. (**b**) The collateral skeletons are shown for a male (cyan) and female (orange) mouse at 4–6 months. (**c**) The number of collaterals across all age groups showed a trending increase (*p* = 0.082) with age that was maximal at 12–15 months. (**d**) No overt differences were found based on sex. With an increased number of collaterals, there was an increased average collateral branch length (**e**), which was not different across sexes (**f**). Distribution of the collateral branch length reveals differences between females (**g**) and males (**h**) at different ages. The average tortuosity index of the collateral branches across age groups was not significantly different (**i**), but males showed significantly elevated tortuosity compared to females at 4–6 months of age (**j**). # indicates a significant effect of age, with # *p* < 0.05. * indicates a significant difference between two groups, with * *p* <0.05. Data shown in (**d**,**f**,**j**) as mean ± SEM.

To further assess potential vascular differences during the lifespan of 3xTg-AD mice, we examined the collateral vessels linking the MCA to the ACA and PCA (located in yellow area, Figure 8a). An example of the skeletonized collateral vessels is shown for two mice at 4–6 months in Figure 8b. The number of collateral vessels in each age group demonstrates a trending increase in numbers with age (*p* = 0.082, one-way ANOVA, Figure 8c). No significant differences in the number of collaterals between males and females across age groups were found (Figure 8d). The average length of collateral vessels progressively increased with age between 1–3 and 7–9 months, with a significant difference between 1–3 and 7–9 month timepoints (* *p* = 0.037, one-way ANOVA, Figure 8e). By 12–15 months, the average length was decreased to 1–3 months levels. However, sex effects were not found among any of the age groups (Figure 8f). The distribution of the collateral branches by length is shown in females (Figure 8g) and males (Figure 8h) at different ages and reveals variability in distribution between age groups for both sexes. No significant differences in the tortuosity index were found between the age groups (Figure 8i), but a sex-dependent increase in collateral vessel tortuosity for 7 to 9-month-old males was observed compared to females (* *p* = 0.037, 2-way ANOVA, Figure 8j).

## 4. Discussion

Vascular abnormalities in human AD are emerging as a prominent comorbidity that is believed to contribute to neuropathology and ultimately, to cognitive dysfunction with age [31]. Vascular topology is altered within AD patient brains, with string vessels, microbleeds, vessel fragmentation, and other modifications [32,33,34]. Aging leads to a remodeled angioarchitecture that results in decrements in vascular function [35,36]. This confluence of vascular events has been proposed to start as early as decades prior to cognitive manifestations of AD [37]. However, little is known about the temporal evolution of this presumably slow cascade of events involving brain vessels (particularly microvessels) in humans. A variety of AD mouse models provide the ability to investigate these temporal events in greater detail. We have recently summarized the vascular structural and functional alterations reported in many mouse models that mimic the features of AD [38].

One popular AD mouse model is the 3xTg-AD mouse, which was developed by combining mutant hAPP (Swedish), PSEN1 (MM146V), and tau (P301L) transgenes [19]. Like human AD, the 3xTg-AD mouse exhibits Aβ and tau pathologies with advancing age, coinciding with behavioral deficits (See [39]). Furthermore, emerging evidence, as seen with molecular biology [17,40,41] and cerebrovascular function [21,42,43], points to early development of dysregulated cerebral blood flow, vascular permeability, and angiogenesis in the 3xTg-AD mouse model. Thus, the current study focused on a remaining knowledge gap: the structural remodeling of the pial arteries and microcirculation that precedes and/or accompanies AD pathology. Although a previous study utilized cerebrovascular corrosion cast and µCT imaging in male mice [16], there has not been a systematic temporal examination of the vascular topology over the lifespan of both male and female 3xTg-AD mice.

**Figure 9:**
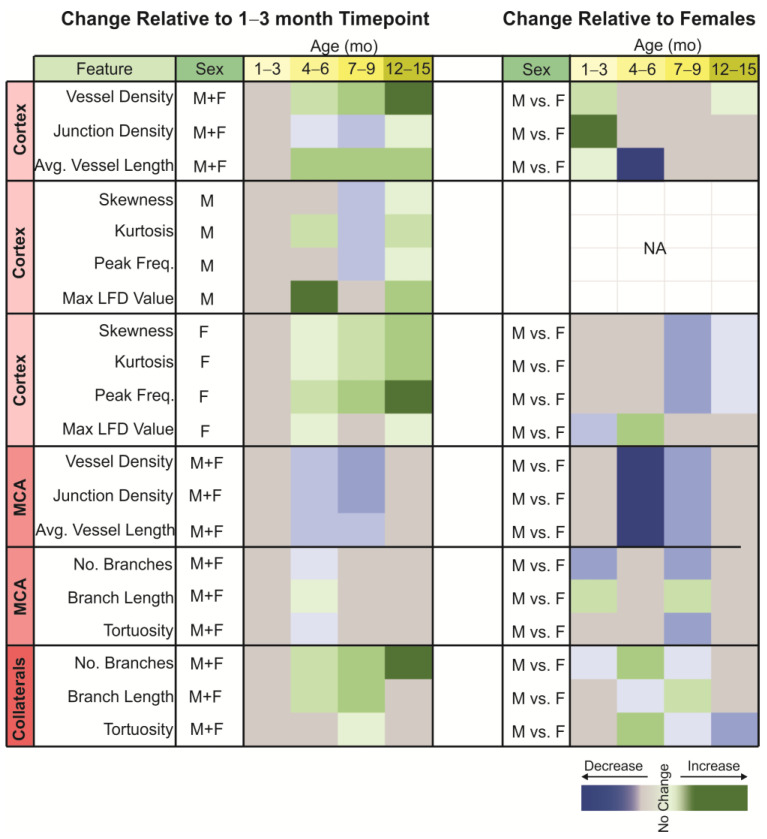
Summary heatmap of relative changes in the 3xTg-AD mouse with increasing age. The heatmap of the change relative to the 1–3 month timepoint illustrates the increasing alterations in vascular topology of the cortex with increasing age. In the middle cerebral artery (MCA), there are decreases in vascular features during mid-life. Relative to female 3xTg-AD mice, male mice predominantly showed decreased vascular features, particularly in the MCA.

We report here for the first time novel vascular features in 3xTg-AD mice from the cortex, and the broad primary findings are that (as summarized in Figure 9): (1) vascular density increases over the 3xTg-AD lifespan, with early (<3 months) increased vessel length that stabilizes later in life, (2) vessel junction density lags that of vessel length, (3) MCA vessel features, including density and junctions, decrease during the first ~60% of the 3xTg-AD mouse lifespan, but then modestly increase by 12–15 months of age, (4) MCA branching and tortuosity are not overtly altered across the lifespan, but a small decrease is observed at 4–6 months of age, (5) there is a progressive increase in ACA/PCA/MCA collaterals across the lifespan associated with increased branch length, (6) cortical vessel complexity via fractal analyses exhibit increasing complexity in female mice with age, but complexity in the male 3xTg-AD mice is more variable. Together these results would suggest that cortical vascular density increases over the lifespan along with collateral anastomoses, but that MCA exhibits a paucity of vessel density early to late in life.

A secondary analysis to determine if there is a sex-dependent change in the lifespan was undertaken (summarized in Figure 9, right panel). Here, our novel findings are that relative to females, males showed: (1) early (<3 months) increased vascular features (density, junctions, length) that disappeared with age, (2) the MCA vessel density was drastically decreased in middle age (4–9 months), but seemingly recovered at 12–15 months, (3) collaterals between the ACA/PCA/MCA were more dynamic with increases and decreases based on age, and (4) male vessel complexity was decreased later in life (>7 months), whereas females exhibited increasing vessel complexity with age. In summary, male 3xTg-AD mice appeared to have decreased vessel features relative to age-matched female mice.

The functional consequences of an altered vascular topology have not been systematically explored in the 3xTg-AD mouse model. The most in-depth cerebrovascular function study was reported by Laranjinha and colleagues, where they examined the neurovascular coupling by modulating neuronal nitric oxide (NO) signaling [20]. The primary finding was that NO signaling evoked a ~50% decrement in cerebral blood flow (CBF) in middle age (12-month-old) 3xTg-AD mice compared to controls. This CBF decrease was still present in old (18-month-old) 3xTg-AD mice. This study suggests that neurovascular coupling is impaired early in life, possibly as the result of an altered angioarchitecture that we observed in our study. The cerebrovascular volume in 3xTg-AD mice is was also decreased as early as 6 months of age and was still present by 11 months, partially attributed to an increase in vascular membranes leading to decreased blood volume [44]. Do et al. [40] confirmed the early age (6 months) reductions in cerebrovascular volume prior to pathological alterations. Pericyte coverage in 3xTg-AD mice exhibited a U-shaped function, with early increased coverage at 6 months, followed by a dramatic decline at 9 months, and then an even larger increase by 12 months of age [17]. This dynamic temporal change in pericyte coverage is intriguing, as it may mirror the changes in the cortical vessel length and decrements in MCA vessel features, as we report (Figure 3, Figure 6 and Figure 7).

Several therapeutic and interventional strategies have been tested and proposed to improve vascular and cognitive health. One such intervention is the effect of exercise in both human subjects and animal models of AD [41,42] (see [43] for extensive review). A prolonged exercise paradigm (6 months) in 3xTg-AD mice found blunting of the AD-associated cognitive decline that were moderately associated with improved synaptic function [45]. More recently, Kim and colleagues [46] using an even more extensive exercise protocol, demonstrated improved cognitive function that was associated with decreased amyloid plaque burden, reduced neuroinflammation, and increased neurogenesis in the hippocampus. In the 3xTg-AD mouse, there have been no direct studies reporting a linkage between vascularity and exercise, although this has been clearly demonstrated in elderly AD subjects [47]. However, a lack of beneficial effects from exercise have also been reported in AD patients and in other mouse models of AD [48,49]. Clearly, additional research is needed to provide a strong link between altered vascular topology and function and how interventions may improve delivery via an enhanced vascular network.

The current study has several limitations. We did not investigate vascular measures from wild-type mice from the same genetic background (i.e., B6129SF2/J). However, we believe that the contribution of the aging process alone to the pathology in the wild-type strain (≤15 months) is relatively minimal to negligible for the overall health of the brain. Previous publications demonstrated that brain volume [50,51,52], synaptic function [21], learning/memory [18,53], and cerebral blood flow [20] are not overtly affected by aging in the wild-type mice, which contrasts with the development of AD pathology associated with memory loss in the 3xTg-AD mouse [18]. Further, we have recently reported that the commonly used C57BL/6N mice [54] do not exhibit age-related cerebrovascular endothelial function at 15 months of age, which contrasts with decreased function in aging 3xTg-AD mice. Moreover, our ongoing and unpublished work on C57BL/6N mice using vessel painting revealed no significant differences in vessel density across ages (4, 12, and 24 months) and between sexes.

The angioarchitectural approach we utilized in the current study was cross-sectional in design, thus limiting the assessment of vascular development over time in this 3xTg-AD model. Approaches such as two-photon imaging through cranial windows or via implanted miniscopes would allow a more temporal observation within this mouse design, as we have done in brain injury models [55], but it is limited by the region under assessment. We also did not examine vascular function, which limits our ability to link structure–function relationships that may develop with increasing age. Similarly, we did not undertake any behavioral experiments to determine how increasingly altered vessel morphology drives behavioral decrements.

Finally, we did not undertake any histopathological appraisals in light of a recent study that extensively phenotyped the histopathology of the 3xTg-AD mouse model, including Aβ and tau deposition [56]. Briefly, they observed cortical Aβ deposition only in the subiculum at advanced age (18 months), which was significantly driven by aging females. The alterations in cortical vascular topology that we report are likely not directly related to tissue Aβ or tau deposition. Soluble and insoluble cortical tissue Aβ fractions in the 3xTg-AD mouse are elevated at 18mo, whereas cortical tau levels remain relatively constant across the lifespan [56]. Herein, we posit that the early and sex-dependent changes in the cortical vasculature are likely not a direct consequence of Aβ or tau pathology. A similar conclusion was noted by Quintana and colleagues (2021) that emerging vascular morphology is not a consequence of Aβ or tau deposition [16]. Furthermore, the interaction between vessels and constituents of the neurovascular unit (astrocytes, pericytes, and neurons) in the 3xTg-AD and other AD mouse models remains to be elucidated.

## 5. Conclusions

In summary, our evaluation of the cerebrovasculature of the 3xTg-AD mouse model showed that cortical vascular density increases over the lifespan, along with increased territorial anastomosis (ACA/PCA/MCA). Modest sex differences were found, but were predominately localized to male mice, albeit these changes tended to be phasic. The 3xTg-AD angioarchitectural features differ from that of other AD models in which often, either no change or decreased vessel density is reported. The 3xTg-AD mouse model has been reported to have decreased brain metabolism, and the increased vessel density may reflect compensatory measures to provide adequate nutrients. Thus, the 3xTg-AD mouse model of AD is an excellent model to investigate the vascular consequences of increased Aβ and tau pathology and can drive derivation of hypotheses relevant to human AD subjects.

## Data Availability

The data presented in this study are available on request from the corresponding author.

## Supplementary Materials

The following supporting information can be downloaded at: https://www.mdpi.com/article/10.3390/biomedicines10081967/s1, File S1: Macro to run Skeletonize and Analyze Skeleton programs to analyze the patterns of MCA branches.
